# Performance of a novel reusable pediatric pulse oximeter probe

**DOI:** 10.1002/ppul.24295

**Published:** 2019-03-25

**Authors:** Carina King, Tisungane Mvalo, Kristen Sessions, Iain Wilson, Isabeau Walker, Beatiwel Zadutsa, Charles Makwenda, Tambosi Phiri, Nicholas Boyd, Mike Bernstein, Eric D. McCollum

**Affiliations:** ^1^ Institute for Global Health University College London London UK; ^2^ Department of Public Health Sciences Karolinska Institutet Stockholm Sweden; ^3^ University of North Carolina Project Malawi Lilongwe Malawi; ^4^ Department of Pediatrics University of North Carolina Chapel Hill North Carolina; ^5^ Lifebox Foundation London UK; ^6^ Great Ormond Street Hospital London UK; ^7^ Institute for Child Health University College London London UK; ^8^ Parent and Child Health Initiative Lilongwe Malawi; ^9^ King's Sierra Leona Partnership Freetown Sierra Leone; ^10^ Physio Monitor Llt San Ramon California; ^11^ Department of Pediatrics, Eudowood Division of Pediatric Respiratory Sciences Johns Hopkins School of Medicine Baltimore Maryland; ^12^ Department of International Health Johns Hopkins Bloomberg School of Public Health Baltimore Maryland

**Keywords:** hypoxemia, LMIC, pediatric, pulse oximeter

## Abstract

**Objective:**

To assess the performance of reusable pulse oximeter probe and microprocessor box combinations, of varying price‐points, in the context of a low‐income pediatric setting.

**Methods:**

A prospective, randomized cross‐over study comparing time to biologically plausible oxygen saturation (SpO_2_) between: (1) Lifebox LB‐01 probe with Masimo Rad‐87 box (L + M) and (2) a weight‐appropriate reusable Masimo probe with Masimo Rad‐87 box (M + M). A post hoc secondary analysis comparison with historical usability testing data with the Lifebox LB‐01 probe and Lifebox V1.5 box (L + L) was also conducted. Participants, children aged 0 to 35 months, were recruited from pediatric wards and outpatient clinics in the central region of Malawi. The primary outcome was time taken to achieve a biologically plausible SpO _2_ measurement, compared using *t* tests for equivalence.

**Results:**

We recruited 572 children. Plausible SpO_2_ measurements were obtained in less than 1 minute, 71%, 70%, and 63% for the M + M, L + M, and L + L combinations, respectively. A similar pattern was seen for less than 2 minutes, however, this effect disappeared at less than 5 minutes with 96%, 96%, and 95% plausible measurements. Using a ±10 second threshold for equivalence, we found L + M and M + M to be equivalent, but were under‐powered to assess equivalence for L + L.

**Conclusions:**

The novel reusable pediatric Lifebox probe can achieve a quality SpO_2_ measurement within a pragmatic time range of weight‐appropriate Masimo equivalent probes. Further research, which considers the cost of the devices, is needed to assess the added value of sophisticated motion tolerance software.

## 1 INTRODUCTION

Hypoxemia, an oxygen saturation (SpO_2_) less than 90%, is a considerable risk for child pneumonia mortality in low‐middle income countries (LMIC).^1^ Pulse oximetry allows for accurate and noninvasive diagnosis of hypoxemia, but in the absence of oximetry, health providers rely on clinical observations to diagnose severe pneumonia and determine the need for oxygen therapy.^2^ Clinical signs lack accuracy in predicting hypoxemia with pulse oximetry identifying 20% to 30% more hypoxemic cases than clinical signs alone.^3^, ^4^, ^5^ Additionally, identifying clinical signs of severe pneumonia, often by nonphysician clinicians or community health workers, remains inconsistent and unreliable.^6^, ^7^, ^8^, ^9^

Given oxygen availability, universal implementation of pulse oximetry in the 15 highest pneumonia burden countries could avert 148 000 deaths annually.^10^ Despite this, evidence on the uptake of pulse oximetry in LMIC is limited. Available estimates suggests it remains low, ranging from less than 30% to more than 70% across different LMIC settings.^11^, ^12^ There are examples of pulse oximeter implementation being feasible in LMIC settings, including Malawi and Nigeria, and resulting in improved referral decision‐making.^13^, ^14^ Barriers to wider implementation include cost, lack of training and supervision, and lack of robust pulse oximeters and probes. In pediatric populations additional barriers include the lack of a high‐quality, reusable, low‐cost probe that fits all ages of children and is tolerant to movement.^15^

To facilitate routine pulse oximeter implementation and scale‐up, evidence of low‐cost but high‐quality devices being usable in busy clinical settings, typical of many LMIC settings is needed. In response to this call, the Lifebox Foundation led a project to develop a universal pediatric probe in 2016.^16^ Using a human centered design approach to probe development with end‐user usability testing in the United Kingdom, Bangladesh, and Malawi, Lifebox developed a novel probe.^17^ Usability testing found that among 1307 SpO_2_ results, 81% biologically plausible measurements were achieved in less than 2 minutes.^17^

This study builds on this work, assessing how the redesigned Lifebox probe functions when paired with a market leading oximeter microprocessor from Masimo that includes motion and low‐perfusion tolerance software. We aimed to compare this performance with the same Masimo microprocessor and its weight‐appropriate Masimo probe on the same child, to give a direct Lifebox vs Masimo probe comparison. As a secondary objective, we also sought to compare these measurements to historical results that used the redesigned probe with the standard Lifebox V1.5 oximeter microprocessor, which is not enhanced with motion tolerance or low‐perfusion software.

## 2 MATERIALS AND METHODS

We conducted a prospective, randomized cross‐over study comparing (1) the novel Lifebox LB‐01 probe paired with a Masimo Rad‐87 oximeter box (L + M) and (2) a weight‐appropriate reusable Masimo probe paired with the Masimo Rad‐87 oximeter box (M + M; Box 1). The LB‐01 probe used with the Masimo Rad‐87 box was specifically adapted to be compatible for the purposes of this study and is not standard for devices available in the market. Data collection was conducted in May 2018.

Box 1 **Testing protocol for different age and weight categories**

**Table:** 

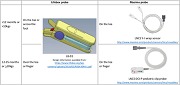

We conducted a post hoc secondary comparison with existing data on the LB‐01 probe paired with the Lifebox V1.5 box (L + L), to explore the added value of motion tolerance capacity. The methods for this study have been reported previously, and data collection was done in February to July 2017.^17^

### 2.1 Settings

Testing was conducted in the central region of Malawi, across three hospitals: Kamuzu Central Hospital (KCH) and Bwaila Hospital in Lilongwe district, and Mchinji District Hospital, in Mchinji district. KCH is a large, tertiary, referral hospital, Bwaila Hospital provides outpatient care only; L + M and M + M data were collected from these sites. Mchinji district hospital provides inpatient and outpatient care, and L + L data were collected from this site.

### 2.2 Recruitment

Children were purposefully recruited using convenience sampling from inpatient and outpatient settings. All children recruited during the cross‐over equivalence study contributed data to the analyses; Figure S1 shows participant inclusion from the historical data. Patients were eligible if they were 0 to 35 months of age excluding those: receiving oxygen therapy; with a nasogastric tube; with a congenital limb malformation; and simultaneously receiving care from a healthcare worker.

### 2.3 Sample size

For the cross‐over study, we were powered to determine equivalence. This required 340 patients to be tested with both L + M and M + M for 80% power to determine equivalence in time to successful measurement within a ±10 second range, with a standard deviation of 40 and a mean time to measurement of 51 seconds. The sample for the L + L testing was based on those meeting eligibility criteria within the existing data set; we did not conduct an a priori power calculation for this analysis.

### 2.4 Data collection

All measurements were conducted by physicians with expertize in pediatric pulse oximetry (TM, EDM, KS, BZ, and NB), following training in the study protocol. For the cross‐over study, two pulse oximetry readings were conducted per child, separated by a 5‐minute washout period, allowing the child to settle and reduce potential measurement bias by the tester. The order in which the probes were used was randomly assigned using a random number generator at the point of testing, within the ODK software used for data collection.^18^

**Figure 1:**
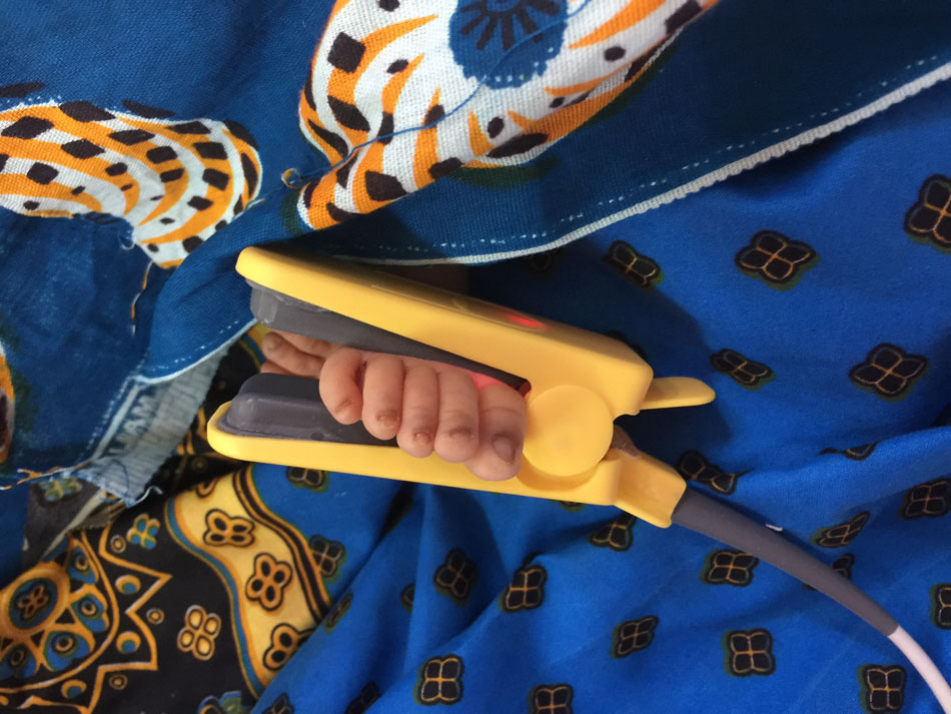
Photograph of the LB‐01 probe in use [Color figure can be viewed at wileyonlinelibrary.com]

The measurement procedure was the same for L + L and the cross‐over study. The tester placed the probe on the foot, toe, or finger of the child, depending on age and weight (Box 1 and Figure 1). An independent observer, a researcher who had received training in the study protocol and was experienced in pulse oximetry but not necessarily clinically qualified, recorded the time from when probe placement was completed to a biologically plausible reading announced by the tester, by them stating “stop.” Biologically plausible was defined as having an age appropriate pulse rate above the approximate 10th centile for age,^19^ and a consistent waveform or quality signal, depending on the oximeter box. The observer noted the condition of the child, location of probe placement, number of adjustments, and any issues during the measurement. Neither the tester, observer, or participants were blinded due to the nature of the measurement; however, randomization was done at the point of testing after a participant was recruited, and the tester could not see the timer during measurements.

### 2.5 Analysis

The primary outcome was the difference in time to a plausible SpO_2_ reading between L + M and M + M, based on a cross‐over design. The primary analysis approach was testing equivalence, defined as ±10 seconds in the mean time to successful measurement (ie, a measurement between 50 and 70 seconds is equivalent to 60 seconds). We chose equivalence, rather than noninferiority, as we did not hypothesize that M + M would necessarily outperform the L + M combination. We deemed ±10 seconds to be a pragmatic range that would not significantly impact routine care in a busy LMIC pediatric setting. We evaluated this through two one‐sided *t* tests, using the ‐*tostt*‐ command in Stata 14.^20^ The comparison between L + M and M + M took into account the paired nature of the data.

A post hoc secondary analysis was conducted to compare the M + M measurements to historical L + L measurements, using the same definition of equivalence. Additionally, we described the median time to SpO_2_, and proportion of SpO_2_ readings within less than 1, 2, and 5 minutes, and conducted a multivariable analysis to examine factors associated with a successful SpO_2_ in less than 1 and 2 minutes, with robust standard errors to account for clustering at the participant level. These models included, probe and box combination, order of the measurement, child's condition, age, and weight. Other potential confounders were investigated for an association between testing rounds and were included if there was a difference. All analyses were conducted using Stata 14.

### 2.6 Ethics

This study was approved by the National Health Science Research Committee of Malawi (ref: 16/4/1570), University College London (ref: 8075/003), and Johns Hopkins (IRB00047406). Verbal consent was obtained from all caregivers.

## 3 RESULTS

### 3.1 Patient characteristics

Overall 572 children were recruited, 232 in L + L testing and 340 in L + M and M + M testing (Table S1). There were significant differences in the presenting diagnoses of children recruited for different testing rounds, with 37% of L + L participants classified as healthy compared with 22% of L + M/M + M (ie, attending for routine postnatal care or vaccination clinics), and 27% of L + L children with acute respiratory infections vs 43% of L + M/M + M children (*P* < 0.001). There was a higher proportion of agitated and crying children in the L + L testing (28%), compared with L + M (12%) and M + M measurements (13%; *P* < 0.001).

### 3.2 Testing procedures

Overall 174 of 340 (51%) of the cross‐over study measurements were randomized to L + M first, and we did not observe any differences in patient characteristics based on randomization order (Table 1). Seventy‐five percent of SpO_2_ measurements were on the child's toe, followed by 23% on the child's foot. As recorded by the independent observer, there was no difference in the number of probe repositions between the different probe and oximeter combinations (0 repositions: 85% M + M, 84% L + M, 80% L + L; *P* = 0.704). There were 13 cases where issues during the measurement were attributed to the Lifebox oximeter box, three to the Masimo oximeter box, seven to the Lifebox probe, and 10 for the Masimo probes (9 = wrap and 1 = pediatric clip). Reported issues were similar between devices and included: poor quality signals, slow or no presentation of SpO_2_ results, and implausibly low pulse rates.

**Table 1:** Description of patient's recruited to the cross‐over study and characteristics according to the order of randomization

Participant characteristics		Overall (N = 340)	M+M first (N = 166)	L+M first (N = 174)	*P* value^a^
Age, median (IQR), mo		6 (0‐16)	6 (1‐14)	6 (1‐13)	0.985
Weight, mean (SD), kg		7.3 (3.1)	7.5 (3.1)	7.2 (3.0)	0.777
Skin color					
Black		336 (99%)	164 (99%)	173 (99%)	0.535
White		3 (1%)	2 (1%)	1 (1%)	
Primary diagnosis					
ARI		145 (43%)	70 (42%)	75 (43%)	0.855
Fever		77 (23%)	40 (24%)	37 (21%)	
Healthy		75 (22%)	34 (20%)	41 (24%)	
Other		43 (13%)	22 (13%)	21 (12%)	
Recruitment location					
Lilongwe (outpatient)		167 (49%)	78 (47%)	89 (51%)	0.443
Lilongwe (inpatient)		173 (51%)	88 (53%)	85 (49%)	
SpO_2_, mean (SD)		96.2 (3.48)	96.1 (3.38)	96.3 (3.59)	0.559
Time to measurement, median (IQR)		33 (23‐66)	33 (23‐69)	33 (23‐58)	0.311

Abbreviations: ARI, acute respiratory infections; IQR, interquartile range; L + M, Lifebox LB‐01 probe with Masimo Rad‐87 box; M + M, a weight‐appropriate reusable Masimo probe with Masimo Rad‐87 box.

^a^ χ^2^ test for binary and categorical variables, and *t* test for continuous variables.

### 3.3 Equivalence

**Figure 2:**
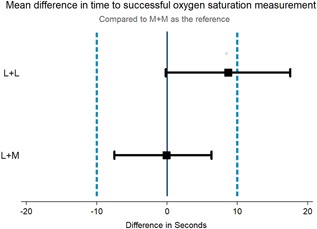
Mean difference in time to biologically plausible oxygen saturation measurement. L + L, Lifebox LB‐01 probe and Lifebox V1.5 box; L + M, Lifebox LB‐01 probe with Masimo Rad‐87 box; [Color figure can be viewed at wileyonlinelibrary.com]

The mean time to L + M measurement was 51.9 seconds (95% confidence interval [CI]: 47.1, 56.7) and 52.5 seconds (95% CI: 47.6, 57.5) for the M + M combination (Figure 2). Using the ±10 second threshold for equivalence, we found L + M and M + M to be equivalent (*P* < 0.001 and 0.002). They were equivalent down to a threshold of ±7 seconds (*P* = 0.003 and 0.033).

The mean time to measurement for the L + L combination was 61.2 seconds (95% CI: 53.3, 69.1)—an average of 8.7 seconds longer than the M + M measurements. We did not have sufficient power to test for equivalence between L + L and M + M measurements.

### 3.4 Time to successful SpO_**2**_ measurement

Table 2 shows the proportion of biologically plausible SpO_2_ measurements for device combinations across age and weight groups. Plausible SpO_2_ measurements were obtained in less than 1 minute, 71%, 70%, and 63% for the M + M, L + M, and L + L combinations, respectively. A similar pattern was seen for less than 2 minutes, however, this effect disappeared at less than 5 minutes. Performance across age and weight groups showed a clear trend favoring M + M for the less than 2‐month age group, with 78%, 62%, and 48% measurements in less than 1 minutes for M + M, L + M, and L + L, respectively. However, by less than 2 minutes this distinction was no longer observed for M + M and L + M (89% and 88%). All device combinations performed better in children more than 10 kg, while performance was mixed when comparing less than 2 months with 2 to 11 months.

**Table 2:** Description of time to reading, comparing the three different probe and device combinations, stratified by age and weight groups

Test round	Total SpO_2_ tests	Biologically plausible SpO_2_ < 1 min, n (%)	95% CI	Biologically plausible SpO_2_ < 2 min n, (%)	95% CI	Biologically plausible SpO_2_ < 5 min, n (%)	95% CI	Time, median (IQR), s
M + M								
Overall	340	240 (71)	65‐75	297 (87)	83‐91	328 (96)	94‐98	33 (23‐66)
0‐2, mo	94	73 (78)	68‐86	84 (89)	81‐95	93 (99)	94‐100	28 (23‐49)
2‐11, mo	137	83 (61)	52‐69	112 (82)	74‐88	127 (93)	87‐96	44 (26‐82)
12‐35, mo	109	84 (77)	68‐85	101 (93)	86‐97	108 (99)	95‐100	29 (22‐57)
<10, kg	271	179 (66)	60‐72	230 (85)	80‐89	260 (96)	93‐98	37 (24‐74)
≥10, kg	68	60 (88)	78‐95	66 (97)	90‐100	67 (99)	92‐100	26 (21‐40)
L + M								
Overall	340	237 (70)	65‐75	306 (90)	86‐93	328 (96)	94‐98	34 (24‐68)
0‐2, mo	95	59 (62)	52‐72	84 (88)	80‐94	92 (97)	91‐99	39 (26‐86)
2‐11, mo	137	91 (66)	57‐73	118 (86)	78‐91	130 (95)	89‐97	38 (27‐69)
12‐35, mo	109	88 (81)	73‐89	105 (96)	92‐99	107 (98)	95‐100	28 (20‐41)
<10, kg	271	177 (65)	59‐71	238 (88)	83‐91	259 (96)	92‐98	38 (26‐73)
≥10, kg	68	59 (87)	76‐94	67 (99)	92‐100	68 (100)	95‐100	25 (19‐35)
L + L								
Overall	232	147 (63)	57‐70	186 (80)	74‐85	221 (95)	92‐98	35 (20‐84)
0‐2, mo	71	34 (48)	36‐60	53 (75)	63‐84	69 (97)	90‐100	65 (31‐113)
2‐11, mo	65	38 (58)	46‐71	49 (75)	63‐85	59 (91)	81‐97	39 (20‐95)
12‐35, mo	96	75 (78)	69‐86	84 (88)	79‐93	93 (97)	91‐99	22 (16‐46)
10, kg	164	93 (57)	49‐64	127 (77)	70‐84	155 (95)	90‐97	44 (20‐100)
≥10, kg	59	48 (81)	69‐90	52 (88)	77‐95	57 (97)	88‐100	23 (16‐46)

Abbreviations: CI, confidence interval; IQR, interquartile range; L + L, Lifebox LB‐01 probe and Lifebox V1.5 box; L + M, Lifebox LB‐01 probe with Masimo Rad‐87 box; M + M, a weight‐appropriate reusable Masimo probe with Masimo Rad‐87 box.

Compared with M + M, the adjusted odds of plausible SpO_2_ measurement in less than 1 minute was 9% (95% CI: 0.65, 1.27) lower for L + M and 16% (95% CI: 0.52, 1.35) lower for L + L (Table 3). Notably, neither of these differences was statistically significant when adjusted for age, weight, child's condition, order of SpO_2_ measurement, presenting diagnosis, and accounting for the clustered nature of the data. Plausible SpO_2_ measurement in less than 1 minute was associated with an age of 12 to 35 months (adjusted odds ratio [aOR]: 3.36; 95% CI: 1.76, 6.42); more than 10 kg (aOR: 3.34; 95% CI: 1.90, 6.26); and being asleep (aOR: 2.36; 95% CI: 1.50, 3.68). Using less than 2 minutes as the outcome showed similar magnitude and direction of associations, except L + M showed a higher but nonsignificant odds of plausible measurement (aOR: 1.28; 95% CI: 0.80, 2.05; Table S2).

**Table 3:** Associations between biologically plausible measurement in less than  1 minute and probe and device combinations, adjusted for confounders

Characteristics	SpO_2_ <1 min	SpO_2_ >1 min	OR (95% CI)	*P* value	aOR (95% CI)^a^	*P* value
Oximeter and probe						
Masimo + Masimo	240	100	1.00		1.00	
Masimo + Lifebox	237	103	0.96 (0.72‐1.28)	0.774	0.91 (0.65‐1.27)	0.573
Lifebox + Lifebox	147	85	0.72 (0.50‐1.05)	0.087	0.84 (0.52‐1.35)	0.477
Testing order						
First measure	393	179	1.00		1.00	
Second measure	231	109	0.97 (0.74‐1.26)	0.796	0.81 (0.58‐1.13)	0.215
Age, mo						
0‐2	165	94	1.00		1.00	
2‐11	212	127	0.95 (0.66‐1.38)	0.791	1.34 (0.81‐2.21)	0.248
12‐35	247	67	2.10 (1.41‐ 3.14)	<0.001	3.36 (1.76‐6.42)	<0.001
Weight, kg						
<10	449	257	1.00		1.00	
≥10	167	28	3.41 (2.15‐5.41)	<0.001	3.34 (1.90‐6.26)	<0.001
Child's condition						
Calm	349	143	1.00		1.00	
Agitated	30	47	0.26 (0.15‐0.44)	<0.001	0.14 (0.08‐0.27)	<0.001
Crying	30	44	0.28 (0.16‐0.48)	<0.001	0.12 (0.07‐0.23)	<0.001
Sleeping	215	54	1.63 (1.12‐2.38)	0.011	2.36 (1.50‐3.68)	<0.001
Child's diagnosis						
ARI	231	122	1.00		1.00	
Fever	147	47	1.65 (1.08, 2.52)	0.020	1.13 (0.71‐1.80)	0.614
Healthy	153	83	0.97 (0.66, 1.43)	0.892	1.05 (0.63‐1.76)	0.838
Other	93	36	1.36 (0.83, 2.24)	0.219	1.22 (0.69‐2.15)	0.503

Abbreviations: aOR, adjusted odds ratio; ARI, acute respiratory infections; CI, confidence interval; SpO_2_, peripheral oxyhemoglobin saturation.

^a^ All variables were included in the multivariable model. Multiple testing within individual children has been accounted for using robust standard errors.

## 4 DISCUSSION

Hypoxemia, a key risk for child mortality, can easily be measured using pulse oximetry, but barriers to widespread implementation in LMIC settings are commonly cited as cost and the lack of devices specifically designed for children in these settings. We found that, independent of the oximeter box, the Lifebox and Masimo probes were equivalent in achieving biologically plausible SpO_2_ measurements within as little as ±7 seconds of one another. However, the Lifebox probe in combination with the Lifebox oximeter box was marginally but inconclusively slower.

Equivalence of the probes is an important finding. The technical specification of the novel universal Lifebox probe used across age ranges has been openly published, allowing any manufacturers to make this design.^21^ It is projected to retail for $25, in comparison to the two probes recommended by Masimo for this age range commercially retailing at approximately $100 to $125 each at the time of publication, plus specialty cables to connect the Masimo probes to the oximeter boxes (≈$140/cable). Similarly, there is a price difference between the Rad‐87 and the Lifebox oximeter box, with Masimo oximeter boxes retailing at ≈$450 to $700 vs $250 for Lifebox. Establishing that a more affordable device, of comparable quality to a market leading oximeter, would challenge the assumption that cost is a barrier to scale‐up.

The overall achievement of biologically plausible SpO_2_ achieved in less than 2 minutes for 90% of L + M measurements shows improvement on usability testing conducted in Malawi, Bangladesh, and the United Kingdom with a range of healthcare providers to develop the LB‐01 probe (81% <2 minutes).^17^ This suggests the LB‐01 probe was being limited in its performance by the software in the Lifebox oximeter box. A key finding was the trend toward the Masimo microprocessor box being quicker, independent of probe, indicating more sophisticated motion tolerance software improves performance. Despite being unable to test equivalence for M + M and L + L measurements, the L + L group were on average the longest measurements. As this patient group was slightly older, healthier and more agitated, it suggests these children may have been more mobile during testing, possibly accounting for the poorer performance. This emphasizes the importance of software that can account for motion, a key challenge that has been highlighted by healthcare providers in small children.^15^

Crucial to innovation in this field is ensuring lower‐cost oximeter boxes designed for LMIC settings are not poor quality. Masimo announced the development of the Rad‐G device, designed specifically for spot‐checking for the LMIC market.^22^ It will be crucial to subject this new device to pragmatic testing in the field, to ensure it maintains the current low‐perfusion and motion tolerant software, which we believe to be important. Other initiatives for low‐cost devices for LMIC settings include smartphone based oximetry.^23^, ^24^ With multiple initiatives, generating clear evidence for policy makers and procurement agencies will be crucial to support implementation and scale‐up. Our testing approach could be expanded to benchmark usability, as continuing to evaluate the added value of novel devices in real‐world settings is as important as laboratory‐based accuracy testing.

An important finding was the difference in device performances according to age. The M + M combination was more successful than L + M combination in children less than 2 months within less than 1 minute, although this effect disappeared within less than 2 minutes. A Y‐sensor was used for this age group in the M + M measurements according to Masimo probe specifications (Box 1), suggesting a difference between Y and clip probe designs in smaller infants. It is important to note that we did not record time to probe placement and all testing was done by experts, therefore, taking this into consideration among healthcare workers with less training may increase the measurement time of the Y‐sensor. We would consider our comparison of M + M to L + M in the children less than 12 months to be conservative, with L + M potentially outperforming if we had included time to probe placement. As spot‐checking needs to be quick and easy, straightforward placement is important and future research should consider healthcare provider preference and technique for site selection and placement with different probes. Additionally, as the majority of pneumonia burden is seen in less than 24‐months old,^25^ a universal design may favor a clip.

We had three key limitations: firstly, the tester in the cross‐over study could not be blinded. The tester may have had an inherent preference for one probe or box over another, either through prior personal experience or experience during the testing. This could have influenced their decision on when to accept a measurement as biologically plausible. We were aware of this potential bias during study design, and decided to randomize at the point of measurement rather than in‐advance to reduce the potential for selective recruitment of participants. In addition, the independent observer served both a pragmatic and quality control role, to reduce non‐standardized testing. The second limitation was the potential for both the child's and the tester's behavior to be modified between the first and second measurement. For example, children may have become calmer on second measurement as they were familiar with the process and tester, or the tester may have modified where and how they chose to place the probe based on their recent experience. Again, we were aware of this in the design stage and included the 5‐minute washout period between measurements, and included standardized locations where the first placement of the probe should be according to age and weight in the protocol. We included the order of measurement in the adjusted analyses, and found that it was not significantly associated with successful measurements, suggesting these biases were not present. Finally, we lacked sufficient power from the historical L + L testing to conduct an equivalence analysis, limiting our ability to make a conclusion for this comparison. A prospective three‐way cross‐over could have overcome this limitation, however, it was beyond the scope of testing at the time.

We found the novel universal reusable pediatric Lifebox clip probe can achieve a quality SpO_2_ measurement within a pragmatically equivalent time as the Masimo reusable Y‐wrap sensor on children less than 10 kg and the reusable Masimo pediatric clip probe on children more than 10 kg. As cost and sustainability is frequently cited as a key barrier to pulse oximeter implementation and scale‐up in LMICs, this is an exciting finding as Lifebox probes are typically available at a fraction of the cost of market leading reusable probes, and requires a single probe for all children rather than multiple specialty designs. Further work is needed to improve motion tolerance in low‐cost oximeter boxes to fully realize the potential of pulse oximetry as a routine point‐of‐care diagnostic for pediatric hypoxemia in low‐resource settings. Additionally, as new devices are released, including multi‐model devices with multiple integrated functions, it will be crucial to continue benchmarking these devices not only on cost and laboratory accuracy, but on real‐world applicability.

## AUTHOR CONTRIBUTIONS

The study was designed by CK, EDM, IWa, IWi, and MB, with input from TM and KS. The data collection tools were developed by CK. Data collection was conducted by KS with supervision from TM and EDM for the cross‐over equivalence, and by BZ, EDM, and NB, with supervision from TP and CM for the historical usability testing. Data cleaning and analysis was conducted by CK. The manuscript was written by CK, TM, and KS with significant input from EDM. All authors read, commented and approved the final manuscript.

## CONFLICT OF INTERESTS

The authors declare that there are no conflict of interests.

## Supporting information

Supplementary information

Supplementary information

Supplementary information
