# Supplementary material for: Performance of a novel reusable pediatric pulse oximeter probe
Source: Pediatr Pulmonol. 2019 Mar 25;54(7):1052–9. doi: 10.1002/ppul.24295 (PMC6591029; doi:10.1002/ppul.24295)
Supplement: Supplementary file 1 — Supplementary information [file PPUL-54-1052-s001.docx]

Supplementary Figure 1: STROBE diagram for participant inclusion from the historical usability testing dataset

Total measurements = 1,307

Exclusion:

- Bangladesh or UK = 830
- Healthcare worker test = 205
- Aged >35 months = 40

Eligible for inclusion = 232

Included in analysis = 232
