# Supplementary material for: Performance of a novel reusable pediatric pulse oximeter probe
Source: Pediatr Pulmonol. 2019 Mar 25;54(7):1052–9. doi: 10.1002/ppul.24295 (PMC6591029; doi:10.1002/ppul.24295)
Supplement: Supplementary file 2 — Supplementary information [file PPUL-54-1052-s002.docx]

**Supplementary Table 1:** Description of children included in the different testing rounds

| **Participant Characteristics** | | **Overall**  **(N = 572)** | **M+M / L+M***  **(N = 340)** | **L+L**  **(N = 232)** | **p-value**** |
| --- | --- | --- | --- | --- | --- |
| Age in months, median (IQR) | | 6 (0 – 16) | 6 (1 – 14) | 8 (0 – 18) | 0.059 |
| Weight in kg, mean (SD) | | 7.3 (3.1) | 7.2 (3.1) | 7.5 (3.2) | 0.212 |
| Skin color | Black  White | 567 (99%)  4 (1%) | 336 (99%)  3 (1%) | 231 (99%)  1 (<1%) | 0.525 |
| Primary diagnosis | ARI | 208 (36%) | 145 (43%) | 63 (27%) | <0.001 |
|  | Fever | 117 (20%) | 77 (23%) | 40 (17%) |  |
|  | Healthy | 161 (28%) | 75 (22%) | 86 (37%) |  |
|  | Other | 86 (15%) | 43 (13%) | 43 (19%) |  |
| Recruitment location | Lilongwe (outpatient) | 334 (37%) | 334 (49%) | - | - |
|  | Lilongwe (inpatient) | 346 (38%) | 346 (51%) | - |  |
|  | Mchinji (inpatient/outpatient) | 232 (25%) | - | 232 (100%) |  |

*The same children were used to test both probe and box combinations, as this was designed as pre-post equivalence study

**Difference between the two rounds of testing, using Chi2 test for categorical and t-test for continuous variables

L+M: Lifebox LB-01 probe with Masimo Rad-87 box; M+M: a weight-appropriate reusable Masimo probe with Masimo Rad-87 box; IQR: Inter-quartile range; SD: standard deviation; ARI: acute respiratory infection.
