# Supplementary material for: Performance of a novel reusable pediatric pulse oximeter probe
Source: Pediatr Pulmonol. 2019 Mar 25;54(7):1052–9. doi: 10.1002/ppul.24295 (PMC6591029; doi:10.1002/ppul.24295)
Supplement: Supplementary file 3 — Supplementary information [file PPUL-54-1052-s003.docx]

**Supplementary Table 2**

Associations between biologically plausible measurement in <2 minute and probe and device combinations, adjusted for confounders

| **Characteristic** |  | **SpO_2_ <2 minute** | **SpO_2_ >2 minute** | **OR (95% CI)** | **p-value** | **aOR (95% CI)*** | **p-value** |
| --- | --- | --- | --- | --- | --- | --- | --- |
| Oximeter and probe | Masimo + Masimo  Masimo + Lifebox  Lifebox + Lifebox | 297  306  186 | 43  34  46 | 1.00  1.30 (0.85, 2.00)  0.59 (0.36, 0.95) | 0.226  0.030 | 1.00  1.28 (0.80, 2.05)  0.70 (0.38, 1.27) | 0.305  0.241 |
| Testing order | First measure | 491 | 81 | 1.00 |  | 1.00 |  |
|  | Second measure | 298 | 42 | 1.17 (0.80, 1.72) | 0.421 | 0.87 (0.55, 1.39) | 0.568 |
| Age | 0-2 months  2-11 months  12-35 months | 221  279  290 | 39  60  24 | 1.00  0.82 (0.51, 1.34)  2.14 (1.21, 3.79) | 0.432  0.009 | 1.00  1.32 (0.69, 2.53)  4.86 (1.90, 12.44) | 0.401  0.001 |
| Weight | <10 kg  >=10 kg | 595  185 | 111  10 | 1.00  3.45 (1.75, 6.79) | <0.001 | 1.00  2.71 (1.18, 6.21) | 0.018 |
| Child’s condition | Calm  Agitated  Crying  Sleeping | 434  50  50  255 | 58  27  24  14 | 1.00  0.25 (0.14, 0.44)  0.28 (0.15, 0.50)  2.43 (1.32, 4.50) | <0.001  <0.001  0.005 | 1.00  0.17 (0.09, 0.32)  0.13 (0.06, 0.26)  2.86 (1.44, 5.66) | <0.001  <0.001  0.003 |
| Child’s diagnosis | ARI | 296 | 57 | 1.00 |  | 1.00 |  |
|  | Fever | 177 | 17 | 2.00 (1.10, 3.67) | 0.024 | 1.38 (0.72, 2.62) | 0.332 |
|  | Healthy | 203 | 33 | 1.18 (0.71, 1.96) | 0.512 | 1.55 (0.78, 3.10) | 0.210 |
|  | Other | 113 | 16 | 1.36 (0.69, 2.68) | 0.374 | 1.35 (0.62, 2.95) | 0.456 |

*All variables were included in the multivariable model. Multiple testing within individual children has been accounted for using robust standard errors. ARI: Acute respiratory infection; SpO_2_: peripheral oxyhemoglobin saturation.
